# Effects of Repeated Heating on Fatty Acid Composition of Plant-Based Cooking Oils

**DOI:** 10.3390/foods11020192

**Published:** 2022-01-12

**Authors:** Zoltan Szabo, Tamas Marosvölgyi, Eva Szabo, Viktor Koczka, Zsofia Verzar, Maria Figler, Tamas Decsi

**Affiliations:** 1 Institute of Nutritional Sciences and Dietetics, Faculty of Health Sciences, University of Pecs, 7621 Pecs, Hungary; zoltan.szabo@etk.pte.hu (Z.S.); verzar.zsofia@pte.hu (Z.V.); maria.figler@aok.pte.hu (M.F.); 2 Institute of Bioanalysis, Medical School, University of Pecs, 7624 Pecs, Hungary; marosvolgyi.tamas@pte.hu; 3 Department of Pediatrics, Clinical Centre, University of Pecs, 7623 Pecs, Hungary; decsi.tamas@pte.hu; 4 Department of Biochemistry and Medical Chemistry, Medical School, University of Pecs, 7624 Pecs, Hungary; koczka.viktor@pte.hu; 5 Doctoral School of Health Sciences, Faculty of Health Sciences, University of Pecs, 7621 Pecs, Hungary; 6 2nd Department of Internal Medicine and Nephrology Centre, Clinical Centre, University of Pecs, 7624 Pecs, Hungary

**Keywords:** repeated heating, fatty acid composition, vegetable oil, frying, linoleic acid, alpha-linolenic acid, *trans* fatty acids, nutritional index

## Abstract

Several polyunsaturated fatty acids are considered to have beneficial health effects, while saturated fatty acids and industrial *trans* fatty acids (TFAs) are linked to negative health consequences. Given the increased formation of TFAs during heating, many studies already investigated compositional changes in oils after prolonged heating or at extremely high temperatures. In contrast, our aim was to measure changes in fatty acid composition and in some health-related indices in edible oils after short-time heating that resembles the conventional household use. Potatoes were fried in palm, rapeseed, soybean, sunflower and extra virgin olive oils at 180 °C for 5 min, and samples were collected from fresh oils and after 1, 5 and 10 consecutive heating sequences. Regardless of the type of oil, the highest linoleic acid and alpha-linolenic acid values were measured in the fresh samples, whereas significantly lower values were detected in almost all samples following the heating sequences. In contrast, the lowest levels of TFAs were detected in the fresh oils, while their values significantly increased in almost all samples during heating. Indices of atherogenicity and thrombogenicity were also significantly higher in these oils after heating. The present data indicate that prolonged or repeated heating of vegetable oils should be avoided; however, the type of oil has a greater effect on the changes of health-related indices than the number of heating sequences.

## 1. Introduction

The fatty acid (FA) composition of edible oils plays an important role in human nutrition. Essential fatty acids (EFAs) cannot be synthesized in the human body and should be taken up with our diet. Both the omega–6 (n–6) EFA, linoleic acid (C18:2n–6, LA) and the omega–3 (n–3) EFA, alpha-linolenic acid (C18:3n–3, ALA) as well as their most important, longer chain derivates, arachidonic acid (C20:4n–6, AA), eicosapentaenoic acid (C20:5n–3, EPA) and docosahexaenoic acid (C22:6n–3, DHA) are considered to have beneficial health effects that can influence several areas of human physiology [1,2,3,4].

The physiologically important unsaturated fatty acids contain double bonds in cis configuration causing a bend of the molecule, while *trans* isomeric fatty acids (TFAs), similarly to saturated fatty acids (SFAs), exhibit linear spatial configuration. Because they use the same enzymes during their metabolism, TFAs can disturb the metabolism of n–3 and n–6 fatty acids. Therefore, the unfavorable health effects of TFAs in the human body can be caused by both their more rigid structure in lipid membranes and by their disturbing effect on fatty acid metabolism [5,6].

Although the first animal experiments showed a positive correlation between TFA intake and some unfavorable cardiovascular effects [7] as well as EFA deficiency [8,9] as early as the 1970s, reports of human data on the adverse effects of hydrogenated oils and TFA intake on cardiovascular health appeared in greater numbers only from the 1990s [10,11,12,13]. Regardless of the dietary source, TFAs can increase plasma concentrations of triacylglycerols, total cholesterol and low density lipoprotein (LDL) [11,14], and lower that of high density lipoprotein (HDL) [11]. They can also increase the risk of myocardial infarction [15] and coronary heart disease [12,16]; however, studies showing the correlation between their putative adverse effect and insulin sensitivity or the risk of developing type 2 diabetes mellitus are rather controversial [17,18,19].

Naturally occurring TFAs are mainly produced in the stomachs of ruminants. The main dietary sources of human TFA exposure, however, are the partially hydrogenated vegetable oils, bakery products and deep-fried fast foods [20]. The ruminant derived TFAs can have different health effects as compared to those from the industrial food sources [21,22].

Deep frying is a very popular food preparation process in several parts of the world [23,24] including Hungary [25], where many popular and traditional dishes are deep-fried. In many households all over the world oils are used not only once but reheated several times, despite reports of serious health hazards related to this procedure [26,27,28,29,30]. During heating, many chemical processes occur, like the oxidation of different fatty acids and triacylglycerols [31], polymer or cyclic compound formation [32,33,34], loss of volatile compounds [32] and the formation of polycyclic aromatic hydrocarbons [35]. During the heating process the polyunsaturated fatty acid (PUFA) content of vegetable oils decreases while that of saturated fatty acids (SFAs) usually increases [36,37].

Different indices can be used, based on fatty acid composition, to characterize the health effects of different foods [38]. The index of atherogenicity (IA) includes the relationship between the sum of the main saturated (i.e., mostly pro-atherogenic) FAs and that of the main unsaturated (i.e., mostly anti-atherogenic) FAs [39]. The index of thrombogenicity (IT) reflects the thrombogenic potential of the foods that contain pro-thrombogenic FAs (i.e., mostly saturated) and anti-thrombogenic (MUFAs, n–3 and n–6 PUFAs) FAs in different ratios [39]. In the early 2000s, two further indices were developed for characterizing the health effects of foods: the hypocholesterolemic/hypercholesterolemic (HH) ratio [40] that describes the relationship between hypocholesterolemic FAs (mainly oleic acid [C18:1n–9, OA] and PUFAs) and hypercholesterolemic FAs (mainly SFAs) as well as the health-promoting index (HPI) [41], which is the inverse of the IA.

While deep-frying, a popular food preparation method worldwide, has already been reported to alter fatty acid composition of vigorously heated oils, our aim was to investigate the changes of fatty acid composition in commonly used edible vegetable oils under conventional household conditions. We also investigated the effect of repeated heating processes and calculated nutritional indices to outline potential changes in their health effects.

## 2. Materials and Methods

Based on various databases of the Food and Agriculture Organization of the United Nations (FAO) [42] and United States Department of Agriculture (USDA) [43], we selected three of the most commonly used oils worldwide (palm, soybean and rapeseed oil) and two more oils that are frequently used in Hungary (sunflower and extra virgin (EV) olive oil). Potatoes and oils were bought in a local hypermarket.

### 2.1. Frying Protocol

**Figure 1:**
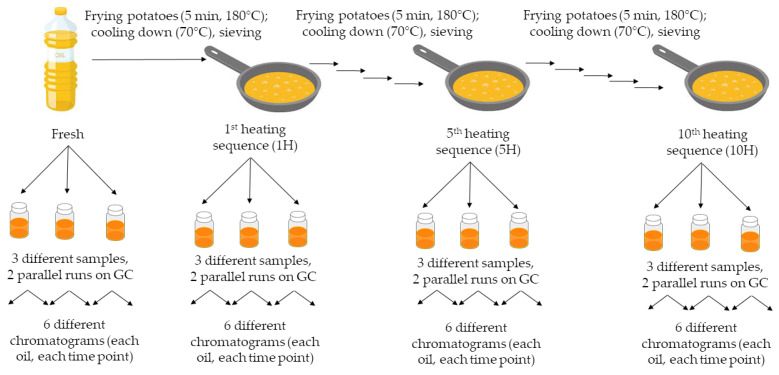
Flow diagram of the frying protocol and sample collection.

The frying procedure was based on The European Food Information Council (EUFIC) [44] and the Hungarian National Food Chain Safety Office (Nébih) [45] protocols with slight modifications. The freshly cut potatoes were fried on 180 °C for 5 min, then potato slices were removed and oils were allowed to cool down to 70 °C. This procedure was repeated ten times after sieving and using freshly cut potatoes. Three different samples were collected from each fresh, unheated oil [Fresh] as well as after the 1st [1H], 5th [5H] and 10th [10H] frying period (Figure 1).

### 2.2. Reagents and Standards

Methanol (Suprasolv^®^ for gas chromatography ECD and FID; Merck, Germany; Cat. No.: 1.06011.2500); n-hexane (Suprasolv^®^ for gas chromatography ECD and FID; Merck, Germany; Cat. No.: 1.04371.1000); pyrogallol (ACS reagent >99%, Sigma-Aldrich, Germany; Cat. No.: 16040-100G-R); acetyl chloride (puriss. p.a., >99.0% (T), Sigma-Aldrich, Germany; Cat. No.: 00990-100ML); 6% K_2_CO_3_-solution (MSURE^®^ ACS, Merck, Germany; Cat. No.: 1049281000) and distilled water were used for sample preparation and analytical measurements.

Peak identification of fatty acid methyl esters was performed by comparison with authentic standards (NuChekPrep; Elysian, MN, USA: GLC–463, GLC–473, GLC–642, GLC–643, GLC–674). We measured the following *trans* isomers: *t*18:1n–9/7, C18:2n–6*tt*, C20:1n–9*t* in the plant-based oils and calculated total TFAs by adding their values together.

### 2.3. Fatty Acid Analysis

The oil samples were stored at −80 °C until chemical analysis. The analysis was performed with a slight modification of our recently published method [46]. In detail, the oil samples were melted at 37 °C, 2.0 μL was transferred into the extraction tube and 3 mL of methanol/hexane (4/1 *v*/*v*) was added. To prevent auto-oxidation, 0.5% pyrogallol was used. During shaking on a vortex mixer, 200 µL acetyl chloride was added. For derivatization, the reaction tubes were placed into a heating block for 1 h at 100 °C. After cooling down, 4.8 mL of K_2_CO_3_ solution (6% *w*/*v*) was added. The samples were centrifuged at 3200 rpm for 10 min at 4 °C. The upper, fatty acid methyl ester containing hexane phase was transferred to vials and analyzed by gas chromatography (GC).

Fatty acid composition was determined by Agilent 6890N GC, which consisted of autosampler 7683B, a flame ionization detector (FID), and cold on column injector. Separation was performed on capillary column DB-23 (60 m × 0.25 mm × 0.25 µm; Agilent J&W Scientific, Folsom, CA, USA).

The inlet temperature gradient was initially kept at 60 °C for 0.1 min, raised to 260 °C at a rate of 114.94 °C·min^−1^ and kept at 260 °C until the end of the measurement. The column oven temperature gradient was initially kept at 50 °C for 2 min, raised to 160 °C at a rate of 40.15 °C·min^−1^, then raised to 184 °C at a rate of 12 °C·min^−1^, and held at 184 °C for 17.5 min. After this the temperature was raised to 190 °C at a rate of 10 °C·min^−1^, and kept there constantly for 17.0 min. Finally, the temperature was raised to 220 °C at a rate of 15 °C·min^−1^, and kept at 220 °C for 5 min.

The carrier gas was H_2_, 2.5 mL/min. Fatty acids were determined from C8:0 to C24:0. The fatty acid composition of each oil sample was determined based on six chromatograms (from two parallel runs of the samples after three independent analytical procedures). Chromatograms were evaluated with Chromeleon 7.1 software (Version 7.1, Thermo Fisher Scientific, Sunnyvale, CA, USA).

### 2.4. Index Calculation

The nutritional indices of fatty acids (IA, IT, HH and HPI) were calculated based on the calculation formulas published by Chen et al. [38].

n–3/n–6 polyunsaturated fatty acid (PUFA) ratio:

$$\frac{\Sigma n-3\mathrm{PUFA}}{\Sigma n-6\mathrm{PUFA}}.$$

Σn–3 PUFA denotes the sum of n–3 polyunsaturated fatty acids (C18:3n–3); Σn–6 PUFA denotes sum of n–6 polyunsaturated fatty acids (C18:2n–6 + C20:2n–6 + C22:2n–6).

Unsaturation index (UI):

UI = 1·(% monoenoics) + 2·(% dienoics) + 3·(% trienoics) + 4·(% tetraenoics) + 5·(% pentaenoics),

monoenoics: C12:1+C15:1n–5+C16:1n–7+C18:1n–7+C18:1n–9+C20:1n–9+C22:1n–9; dienoics:C18:2n–6+C20:2n–6+C22:2n–6; trienoics: C18:3n–3

Index of atherogenicity (IA):

$$\mathrm{IA}=\frac{C12:0+4\cdot C14:0+C16:0}{\Sigma \mathrm{UFA}}.$$

ΣUFA=sum of unsaturated fatty acids (C12:1+C15:1n–5+C16:1n–7+C18:1n–7+C18:1n–9+C20:1n–9+C22:1n–9+C18:3n–3+C18:2n–6+C20:2n–6+C22:2n–6).

Index of thrombogenicity (IT):

$$\mathrm{IT}=\frac{C14:0+C16:0+C18:0}{0.5\cdot \Sigma \mathrm{MUFA}+0.5\cdot \Sigma n-6\mathrm{PUFA}+3\cdot \Sigma n-3\mathrm{PUFA}}.$$

ΣMUFA denotes the sum of monounsaturated fatty acids (C12:1 + C15:1n–5 + C16:1n-7 + C18:1n–7 + C18:1n–9 + C20:1n–9 + C22:1n–9); Σn–6 PUFA denotes the sum of n–6 polyunsaturated fatty acids (C18:2n–6 + C20:2n–6 + C22:2n–6); Σn–3 PUFA denotes the sum of n–3 polyunsaturated fatty acids (C18:3n–3).

Hypocholesterolemic/hypercholesterolemic ratio (HH):

$$\mathrm{HH}=\frac{\mathrm{cis}\;C18:1n9+\;\Sigma \mathrm{PUFA}}{C12:0+C14:0+C16:0}$$

ΣPUFA denotes the sum of polyunsaturated fatty acids (C18:3n–3 + C18:2n–6 + C20:2n–6 + C2:2n–6).

### 2.5. Statistical Analysis

Significant differences were calculated by using one-way repeated measures ANOVA followed by the Bonferroni test. All analyses were conducted by IBM SPSS Statistics for Windows (Version 27.0, SPSS Inc., Chicago, IL, USA). Fatty acids are expressed as a percentage by the weight (*w*/*w*%) of total fatty acids. Data presented in the tables and figures are mean values with standard deviations.

## 3. Results

### 3.1. Fatty Acid Composition of Vegetable Oils

There were substantial differences in the fatty acid composition of the investigated oils (Table 1). The main fatty acids in the palm oil were the saturated C16:0 and monounsaturated OA. We measured the highest SAT and TFA contents in this oil, while the total PUFA content was the second lowest among the oils investigated.

Soybean oil was very rich in both LA and ALA and had the second highest total PUFA content among the investigated oils. Similar to palm oil, MUFA content was low, actually the lowest among the five investigated oils, whereas TFA content was relatively high.

Rapeseed oil had the second highest OA (and therefore total MUFA) as well as the highest ALA contents. Mono- and polyunsaturated fatty acids accounted for more than 90% of total fatty acids in this oil.

Sunflower oil had low SFA and MUFA contents, but LA was present at the highest level, and this oil had the highest PUFA content among the five oils investigated.

EV olive oil had the highest OA and MUFA contents among the plant-based oils and its main PUFA was LA.

### 3.2. Changes in Fatty Acid Composition

#### 3.2.1. Changes in LA Contents during the Heating Sequences

**Figure 2:**
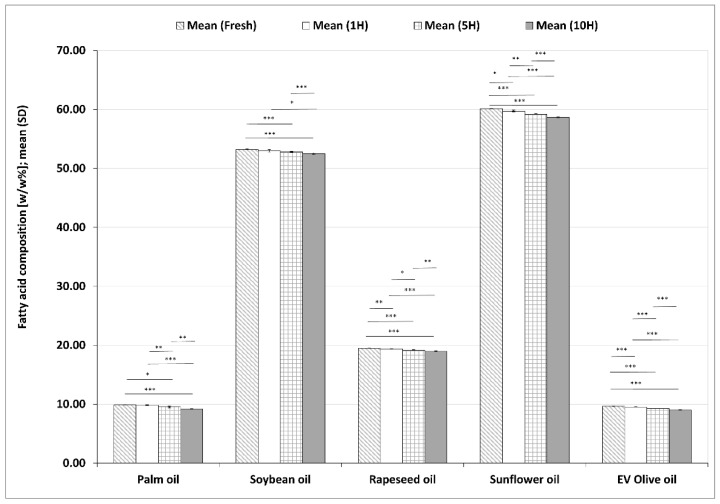
Changes in linoleic acid (LA) values in five different plant-based oils in fresh samples and after one (1H), five (5H) and ten heating sequences (10H). Each column shows the mean (SD) data of six different chromatograms. Asterisks denote statistically significant differences between the different time points in the same oil; *: 0.01 ≤ *p* < 0.05; **: 0.001 ≤ *p* < 0.01; ***: *p* < 0.001. EV olive oil: extra virgin olive oil.

LA contents in the five investigated plant-based oils from fresh form to 10H are summarized in Figure 2. In sunflower oil, LA was the most abundant fatty acid and there was a significant decrease in its value in each sample during the heating process compared to all previous samples. Soybean oil with the second highest LA values showed a significant LA reduction in both 5H and 10H samples compared to fresh oil. The LA values also decreased significantly between 1H and 10H as well as between 5H and 10H samples. The other three vegetable oils investigated, with lower initial LA values, also showed significant and monotonous decreases in LA contents in each investigated period of the heating processes compared to previous periods. After the 10H reheating the reduction of LA compared to the fresh sample was 1.41% in soybean oil, 2.44% in sunflower oil, 2.55% in rapeseed oil, 6.54% in EV olive oil and 6.53% in palm oil, respectively.

#### 3.2.2. Changes in ALA Contents during the Heating Sequences

**Figure 3:**
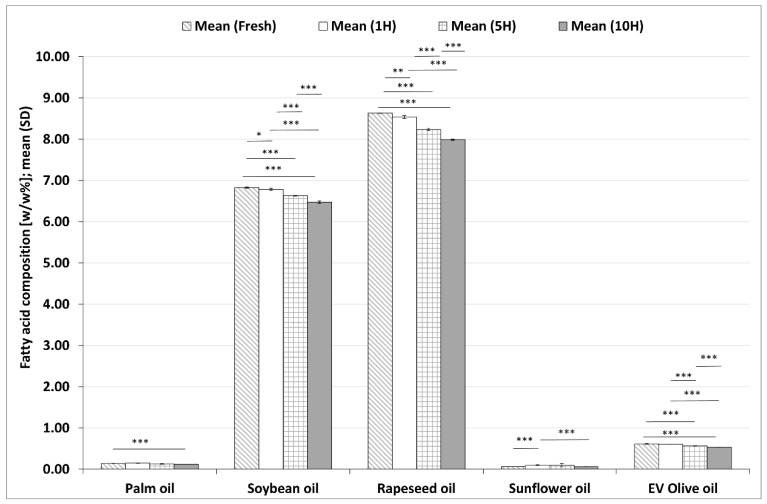
Changes in alpha-linolenic acid (ALA) values in five different plant-based oils in fresh samples and after one (1H), five (5H) and ten heating sequences (10H). Each column shows the mean (SD) data of six different chromatograms. Asterisks denote statistically significant differences between the different time points in the same oil; *: 0.01 ≤ *p* < 0.05; **: 0.001 ≤ *p* < 0.01; ***: *p* < 0.001. EV olive oil: extra virgin olive oil.

ALA contents in the five investigated plant-based oils from fresh form to 10H are summarized in Figure 3. The ALA content of rapeseed oil with the highest, as well as soybean oil with the second highest, ALA values showed a significant decrease in each sample during the heating process compared to all previous samples. Similarly, there were significant decreases in the ALA contents of EV olive oil in each period of the heating process compared to all previous periods, except for values in a fresh sample compared to 1H sample. There was a significant decrease in the ALA contents of sunflower oil between the 1H and the 10H samples. Palm oil ALA contents also decreased significantly after 10 heating sequences compared to fresh samples. After the 10H reheating, the reductions compared to the fresh sample in ALA contents were 2.21% in sunflower oil, 5.12% in soybean oil, 7.40% in rapeseed oil, 11.65% in palm oil and 13.41% in EV olive oil, respectively.

#### 3.2.3. Changes in Total TFA Contents during the Heating Sequences

**Figure 4:**
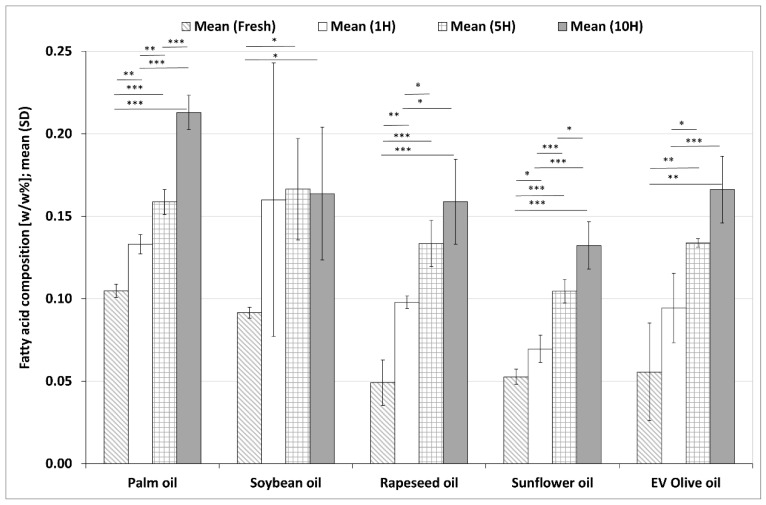
Changes in total *trans* fatty acid (TFA) values in five different plant-based oils in fresh samples and after one (1H), five (5H) and ten heating sequences (10H). Each column shows the mean (SD) data of six different chromatograms. Asterisks denote statistically significant differences between the different time points in the same oil; *: 0.01 ≤ *p* < 0.05; **: 0.001 ≤ *p* < 0.01; ***: *p* < 0.001. EV olive oil: extra virgin olive oil.

Total TFA contents in the five investigated plant-based oils from fresh form to 10H are shown in Figure 4. There were significant increases in total TFA contents in each oil during the heating processes. The extent of the increase between the fresh and 10H samples was 233% in rapeseed oil, 199% in EV olive oil, 151% in sunflower oil, 103% in palm oil and 78.83% in soybean oil, respectively.

All *trans* isomers increased during the heating process in each investigated oils (Table S1). After 10 heating sequences C18:1n–7/9*t* was about 0.1 *w*/*w*% in the investigated oils, while C20:1n–9*t* about 0.06 *w*/*w*%. In almost all samples, the values of *trans* isomers in the 10H samples doubled compared to the corresponding fresh samples.

### 3.3. Changes in Nutritional Indices

#### 3.3.1. Changes in n–3/n–6 Polyunsaturated Fatty Acid Ratios during Heating Sequences

**Figure 5:**
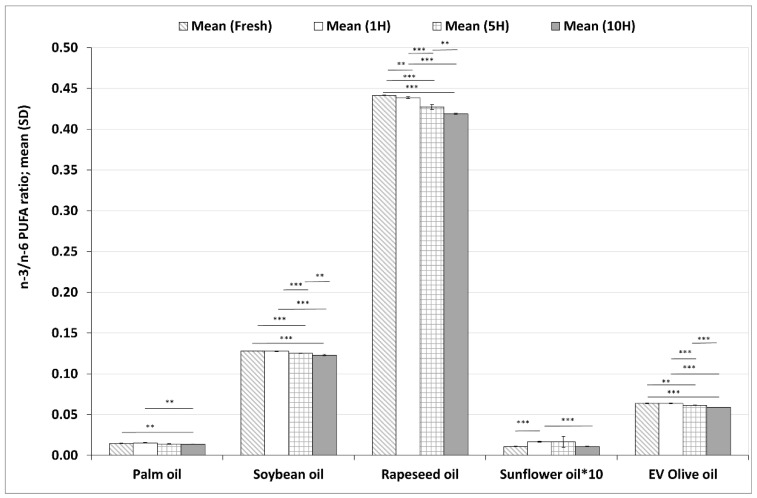
Changes in the n–3/n–6 PUFA ratios in five different plant-based oils in fresh samples and after one (1H), five (5H) and ten heating sequences (10H). Data for sunflower oil are shown at 10× magnification for better visibility. Each column shows the mean (SD) data of six different chromatograms. Asterisks denote statistically significant differences between the different time points in the same oil; *: 0.01 ≤ *p* < 0.05; **: 0.001 ≤ *p* < 0.01; ***: *p* < 0.001. EV olive oil: extra virgin olive oil.

The n–3/n–6 ratios in the five investigated plant-based oils from fresh form to 10H are shown in Figure 5. In rapeseed oil, soybean oil and EV olive oil the n–3/n–6 PUFA ratios were significantly lower at each investigated time point compared to the previous ones (except for fresh sample and after the 1st heating sequence in soybean and EV olive oil). In palm oil, the n–3/n–6 ratio was significantly lower after the 10th heating sequence than in the fresh and 1H samples, whereas in sunflower oil, the n–3/n–6 ratio decreased significantly from the 1H sample and reached its lowest value in the 10H sample.

#### 3.3.2. Changes in the Unsaturation Index (UI) during the Heating Sequences

In the fresh samples, there was a big variation in the UI values of the different oils, with the highest values detected in soybean and sunflower oils and the lowest values in palm oil (Table 2). The UI was about 2.5-fold higher in soybean oil than in palm oil. In soybean oil, after the 10th heating process, UI was significantly lower than in any previous sample. Sunflower oil had the highest UI values as a fresh sample, and it was significantly decreasing during the heating processes. Similar to soybean oil, after 10H, the UI value was significantly lower in sunflower oil than in any other of the previous samples. In the other investigated oils, UI values decreased significantly and reached the lowest levels after the 10th heating sequence. The biggest reduction in UI from fresh to 10H sample was seen in palm oil (2.95%) and the smallest in soybean oil (1.32%) and sunflower oil (1.36%).

#### 3.3.3. Changes in the Index of Atherogenicity (IA) during Heating Sequences

There were big differences in IA values in the investigated fresh oils with the highest value in palm oil and the lowest values in rapeseed oil (Table 2). The difference in IA values between these two oils was not less than 18-fold. In each investigated plant-based oil, IA values increased uniformly during heating and reached the highest levels after the 10th heating process. In each oil the IA value of the 10H sample was significantly lower than those of the fresh oils and the 1H samples. The highest increase between the fresh and 10H samples was detected in sunflower oil (6.76%) while the lowest in EV olive oil (3.37%).

#### 3.3.4. Changes in the Index of Thrombogenicity (IT) during the Heating Sequences

IT values were very variable among the different fresh plant-based oils. Similar to IA, palm oil had the highest value and rapeseed oil the lowest. The difference in the IT values of these two fresh oil samples was more than 20–fold. In each oil, IT values were significantly higher after the 10th heating sequence than in the previous samples. The highest increase between the fresh and the 10H samples was observed in rapeseed oil (7.95%) while the lowest in EV olive oil (3.76%).

#### 3.3.5. Changes in the Hypocholesterolemic/Hypercholesterolemic Ratio (HH) during the Heating Sequences

Similar to the previous indices, the HH ratio values were very diverse among the investigated fresh oils. The difference between the highest (in rapeseed oil) and lowest values (palm oil) was more than 13-fold. In all plant-based oils we saw a uniformly and monotonously decreasing value of HH ratio during the heating processes, with the lowest values after the 10th heating sequence in each oil. The lowest decrease was found in EV olive oil (3.24%), while the highest was found in sunflower oil (5.79%).

## 4. Discussion

During deep-frying, several changes occur that can affect the flavor, color and texture of deep-fried food as well as their nutritional quality. In the frying oil free fatty acid, peroxide, di- and polymer content increases, while the total unsaturated fatty acid content decreases [32]. In the present study we focused on the total fatty acid composition of commonly used plant-based oils and fats and found significant changes in the availability of important dietary fatty acids during the frying process.

In fresh form, there were substantial differences between the fatty acid compositions of the investigated oils. The present data were consistent with the literature by showing that palm oil is mainly composed of palmitic acid (C16:0) and OA [47], whereas the main fatty acids in soybean oil are OA and LA [36,48,49,50,51,52,53,54], in rapeseed oil OA, LA and ALA [36,48,52] and in sunflower oil [36,48,54,55,56] and olive oil [37,48,52,54,55,56] LA and OA.

In the present study, after 10 heating sequences at 180 °C, the TFA values in each oil were significantly higher than in the fresh samples, with the highest increase detected in rapeseed oil (223%, *p* < 0.001). However, even after significant increases the TFA values were still low and remained under 0.25 *w*/*w*%. TFAs certainly form under extreme circumstances, especially during heating at high temperatures for a long time, but studies are controversial with respect to the extent of TFA formation. Heating of the analytic standards triolein and trielaidin [57] at a relatively low temperature (160 °C) for 2 h resulted in the formation of *trans* isomers from the cis OA; whereas addition of antioxidants to the reaction reduced TFA formation. Time- and temperature-dependent cis/*trans* isomerization of OA to elaidic acid (C18:1n–9*t*) was also reported from the same study. Heating however destabilized the double bond regardless to its configuration, so the concentration of *trans* elaidic acid also decreased to~50% after heating at 220 °C for 5 h, and was accompanied by the formation of ~0.5% cis OA [57].

In another study with vegetable oils [50], TFA was only found in unheated hydrogenated but not in non-hydrogenated soybean oil. Heating had no effect on the formation of TFAs even at high temperature (220 °C) for long time (24 h). Similarly, neither heating nor frying induced significant formation of TFAs in corn oil at 170 °C [58]. In contrast, Casal et al. [37] found increasing TFA values during the heating process in EV olive oil as well as in the blend of refined and virgin olive oil and sunflower oil. Although the TFA content of these oils after a longer time of frying (15 to 27 h) did not reach 0.5% of total fatty acids (except for commercially blended refined and virgin olive oil: 0.58% after 27 h), there was a highly significant positive correlation between TFA values and frying time in case of each oil. In refined olive pomace oil, only a small increase of TFA levels, about 1.6% of total fatty acids could be measured even after 60× frying process [59]. However, under more extreme circumstances (at 240 °C for 12 h), higher *trans* formation was observed and TFAs reached not less than 7.7% of total fatty acids [49].

Bhardwaj et al. [23] demonstrated that both heating and frying for 30 min increased the TFA content of each investigated plant-based oil even at a lower temperature (180 °C), whereas higher values were found at higher temperatures and after re-heating/re-frying. In this study, partially hydrogenated vegetable oil contained 13% TFAs in fresh form, and after the heating or frying process it reached 18.70% and 18.88%, respectively. The type of the frying process also influences TFA formation in vegetable oil: deep-frying can lead to higher TFA formation than stir-frying or pan-frying [48]. As a summary, studies showed that the TFA formation can depend on several factors, like the type of oil [23,31,34,37,48,50,60], temperature [23,34,49,50] and time of heating [23,31,34,37,48,50,58,59,60], as well as the mode of cooking (e.g., heating, frying, stir-frying) [23,48,52,58]. Results of the present study also showed that cis-*trans* isomerization occurs even at a lower temperature and with a shorter exposure to frying. Although TFA contents increased continuously with ongoing heating periods, this effect seems to be negligible from the physiological point of view. The World Health Organization (WHO) [61,62], the European Food Safety Authority (EFSA) [63] and the American Heart Association (AHA) [64] recommended that total TFA intake should be as low as possible, but less than 1% of total energy intake. Under the examined conditions of investigated oils (<180 °C; <10× use) TFA intake with fried food remains well below this value: the highest observed TFA value in the oils investigated was found in palm oil to be 0.21 *w*/*w*% after ten heating periods.

According to former studies, the TFA content can be very different among various oil types: plant based oils contain less than 0.5% [31,37,48,49,60], or values even under the detection limit [50,52], whereas refined and partially hydrogenated oils can reach as high as 13.9 *w*/*w*% TFA [23] in their fresh forms. In the present study, fresh form of all investigated oils contained only very small amounts of TFAs (lowest: 0.05 *w*/*w*% in rapeseed oil, highest: 0.1 *w*/*w*% in palm oil).

In the present study, the EFA content of each vegetable oil decreased significantly in the course of heating and there was a steady decline of LA content in each oil after the heating periods. Sunflower oil had the highest LA values in fresh form and the LA loss was 1.46 g/100 g after the 10th heating period. Fresh rapeseed oil had the highest ALA content among the five investigated oils, and the highest ALA loss was also observed here (0.63 g/100 g). Although EV olive oil contained only a small amount of ALA, the largest percentage drop in ALA (13.4%) was observed in this oil after the 10th heating period. The decline in LA and ALA values during the heating process of vegetable oils is much less clearly characterized than the rise in TFA values. This decline in LA values was shown in avocado [60], canola [47], corn [54], EV olive [37], hemp [36], lupin [36], oat [36], olive [37,54,60,65], palm [47], rapeseed [34,36], soybean [49,50,53,54] and sunflower oils [34,36,37,54,65,66]. In contrast, Cui et al. [48] found significantly higher LA values after stir-frying and/or pan-frying in peanut, soybean, rapeseed, sunflower, rice bran and linseed oils than in their fresh forms, while in corn, olive and peony seed oils LA values decreased after frying. ALA values also decreased in the course of heating/frying [34,37,47,49,50,53,54,66] except for some studies with soybean [36], avocado [60], corn [48], rice bran [48] and sunflower seed oils [48].

Unsaturated fatty acids are less stable at higher temperatures because cis double bonds can be saturated, isomerized into *trans* configuration or other oxidation processes may occur. Therefore, vegetable oils rich in the physiologically more beneficial MUFAs and PUFAs can be more susceptible to heating-related degradation than other fats rich in saturated fatty acids. During heating or frying an increase in SFA values can be measured in a time-dependent manner. In the present study, SFA values increased significantly (data not shown) in the course of heating; this finding is in accordance with the results of previous studies [23,36,37,48,54,60,65,66]. This increase in SFA values seems to be more consequent than the decrease in MUFAs and PUFAs, although some studies found divergent results [49,50,67]. In the present study after the 10th heating sequence the values of LA and ALA decreased significantly (*p* < 0.001) in each oil compared to their fresh samples, while TFA (palm, rapeseed, sunflower oil: *p* < 0.001; EV olive oil: 0.001 ≤ *p* < 0.01; soybean oil: *p* < 0.05) and SFA values increased significantly. These results suggest, that in the course of frying the PUFA loss can be explained partly by the cis-*trans* isomerization and partly by the saturation of these double bonds.

Many indices were developed to characterize the nutritional effects of different foods; the first ones were IA and IT, developed in the early 1990s. IA shows the relationship between the main pro-atherogenic saturated fatty acids (C12:0, C14:0 and C16:0) and the anti-atherogenic unsaturated fatty acids. IT characterizes the relationship between the pro-thrombogenic saturated fatty acids (C12:0, C14:0 and C16:0) and the anti-thrombogenic MUFAs, n–3 and n–6 PUFAs. As heating and frying result in a decrease in the MUFA and PUFA values as well as in an increase in SFA and TFA values, these indices will also change into a more atherogenic and thrombogenic direction. Among different food preparation methods, frying can increase IA and IT values to the greatest extent [68,69,70,71]; however, the nature of the oils used during the processes can also greatly influence these values [51,55,56,68,72,73,74]. An experimental study showed that frying at lower temperature, in small amounts of oil and for a short time resulted in the highest IA and PUFA/SFA values [75], so the nutritional quality of fried food could be optimized with careful preparation. Not only frying, but other techniques depending on heat exposure can increase IT values and may also influence the n–3/n–6 PUFA ratios [76]. In the present study a similar trend was shown; after 10 heating sequences all the investigated oils had significantly increased IA and IT values representing less favorable health effects.

The two families of PUFAs can have opposite effects on cardiovascular health; PUFAs belonging to the n–3 family (mainly EPA and DHA) are beneficial, while n–6 PUFAs (mainly AA) may have adverse health effects [77]. Therefore, the n–3/n–6 PUFA ratio can be used for the potential cardiovascular (and health) effect of foods. In the present study, the n–3/n–6 PUFA ratios were declining in each oil during heating sequences, in concert with previous findings on the effect of frying on the n–3/n–6 PUFA ratios in different foods and oils [51,55,56,68,69,70,74,78,79,80].

The HH index is used for characterization of the effect of the fatty acid composition of foods on blood cholesterol levels. OA and PUFAs have hypocholesterolaemic, while SFAs hypercholesterolaemic effects, so a decrease in HH indicates less beneficial cardiovascular effects. Heating decreased the HH ratio in each plant-based oil investigated in the present study. In contrast, a previous study didn’t find lower HH ratio in tilapia after frying [70], while in mussels the HH ratio was even increased compared to fresh samples [80]. To the best of our knowledge, no previous studies have investigated the effect of heating on the HH ratio of oils, only in fried foods.

Previous studies have examined the change of only one or two indices, while in the present study the (five) most important nutritional indices were calculated for each oil at each time point and each index has changed in a less favorable direction as a result of heating. The most stable oil, with the smallest changes was EV olive oil, while the most susceptible oils with the greatest changes in nutritional index values were sunflower and rapeseed oils.

## 5. Conclusions

In this study, repeated heating of vegetable oils resulted in a significant decrease in the nutritionally mostly beneficial polyunsaturated fatty acid values, while values of *trans* isomers and saturated fatty acids with mostly unfavorable nutritional effects significantly increased. Heating also caused a deterioration in several health-related biochemical indices, but the type of oil had a greater effect on the changes of the values of these indices than the number of heating sequences. These findings may support the recommendation that oils rich in PUFA and low in SFA values should be chosen for food preparation, frying should be performed at lower temperatures and multiple reheating should be avoided.

## Figures and Tables

**Table 1:** Fatty acid composition of vegetable oils in fresh, unheated samples.

	Palm Oil		Soybean Oil		Rapeseed Oil		Sunflower Oil		EV Olive Oil	
	Mean	SD	Mean	SD	Mean	SD	Mean	SD	Mean	SD
Saturated fatty acids										
C8:0	**0.01**	0.00	**0.01**	0.00	**0.00**	0.00	**0.01**	0.00	**0.01**	0.00
C10:0	**0.01**	0.00	**0.00**	0.00	**0.01**	0.00	**0.00**	0.00	**0.00**	0.00
C12:0	**0.15**	0.00	**0.01**	0.00	**0.01**	0.00	**0.00**	0.00	**0.00**	0.00
C14:0	**0.91**	0.01	**0.08**	0.00	**0.05**	0.00	**0.08**	0.00	**0.02**	0.01
C16:0	**42.14**	0.10	**10.42**	0.05	**4.34**	0.01	**6.29**	0.01	**14.53**	0.07
C18:0	**4.60**	0.02	**4.15**	0.03	**1.64**	0.00	**3.35**	0.00	**2.47**	0.01
C20:0	**0.40**	0.00	**0.37**	0.00	**0.56**	0.00	**0.25**	0.00	**0.43**	0.01
C22:0	**0.07**	0.00	**0.42**	0.00	**0.30**	0.00	**0.75**	0.01	**0.12**	0.01
C24:0	**0.08**	0.00	**0.15**	0.01	**0.15**	0.00	**0.27**	0.00	**0.07**	0.01
SAT	**48.56**	0.09	**15.78**	0.08	**7.18**	0.01	**11.10**	0.02	**17.83**	0.08
Monounsaturated fatty acids										
C16:1n–7	**0.16**	0.00	**0.10**	0.00	**0.20**	0.00	**0.12**	0.00	**1.49**	0.01
C18:1n–9	**40.34**	0.07	**22.30**	0.03	**59.98**	0.03	**27.65**	0.01	**66.87**	0.12
C18:1n–7	**0.70**	0.00	**1.37**	0.00	**3.10**	0.01	**0.71**	0.00	**3.20**	0.00
C20:1n–9	**0.15**	0.00	**0.21**	0.00	**1.20**	0.00	**0.15**	0.00	**0.29**	0.01
C22:1n–9	**0.00**	0.00	**0.01**	0.00	**0.10**	0.00	**0.00**	0.00	**0.00**	0.00
MUFA	**41.35**	0.08	**23.99**	0.03	**64.58**	0.03	**28.63**	0.01	**71.85**	0.10
*Trans* fatty acids										
*t*18:1n–9/7	**0.08**	0.00	**0.04**	0.00	**0.03**	0.01	**0.03**	0.00	**0.05**	0.02
C18:2n–6*tt*	**0.01**	0.00	**0.01**	0.00	**n.d.**	-	**0.00**	0.00	**n.d.**	-
C20:1n–9*t*	**0.02**	0.00	**0.05**	0.00	**0.02**	0.01	**0.02**	0.00	**0.01**	0.01
TFA	**0.10**	0.00	**0.09**	0.00	**0.05**	0.01	**0.05**	0.00	**0.06**	0.03
Polyunsaturated fatty acids										
C18:2n–6	**9.84**	0.01	**53.23**	0.09	**19.49**	0.01	**60.12**	0.02	**9.64**	0.02
C18:3n–3	**0.14**	0.00	**6.82**	0.01	**8.63**	0.01	**0.07**	0.00	**0.62**	0.00
C20:2n–6	**0.00**	0.00	**0.07**	0.00	**0.07**	0.00	**0.04**	0.01	**0.00**	0.00
n–6 PUFA	**9.84**	0.01	**53.32**	0.09	**19.56**	0.01	**60.16**	0.02	**9.65**	0.02
n–3 PUFA	**0.14**	0.00	**6.82**	0.01	**8.63**	0.01	**0.07**	0.00	**0.62**	0.00

Fatty acid values are presented as *w*/*w*% of total fatty acids; each data is calculated from six different chromatograms, in mean (SD); n.d. denotes under detection limit. EV olive oil: extra virgin olive oil, SAT: sum of saturated fatty acids, MUFA: sum of monounsaturated fatty acids, TFA: sum of *trans* fatty acids, n–6 PUFA: sum of n–6 polyunsaturated fatty acids, n–3 PUFA: sum of n–3 polyunsaturated fatty acids.

**Table 2:** Changes in nutritional indices in five different plant-based oils in fresh samples and after one (1H), five (5H) and ten heating sequences (10H).

	Fresh	1H	5H	10H
Unsaturation index [UI]				
Palm oil	61.462 ^aA^ (0.104)	61.405 ^BC^ (0.458)	60.548 ^aBD^ (0.274)	59.646 ^ACD^ (0.275)
Soybean oil	151.093 ^AB^ (0.206)	150.618 ^C^ (0.577)	149.923 ^AD^ (0.210)	149.100 ^BCD^ (0.229)
Rapeseed oil	129.597 ^aA^ (0.022)	129.123 ^BC^ (0.125)	128.111 ^aBD^ (0.153)	127.422 ^ACD^ (0.081)
Sunflower oil	149.138 ^aAB^ (0.039)	148.524 ^abC^ (0.336)	147.91 ^AbD^ (0.092)	147.112 ^BCD^ (0.120)
EV Olive oil	92.988 ^AB^ (0.114)	92.732 ^CD^ (0.057)	92.290 ^ACE^ (0.036)	91.643 ^BDE^ (0.117)
Index of atherogenicity [IA]				
Palm oil	0.895 ^ab^ (0.004)	0.898 ^cA^ (0.013)	0.915 ^acB^ (0.008)	0.937 ^bAB^ (0.011)
Soybean oil	0.128 ^AB^ (0.001)	0.129 ^a^ (0.002)	0.131 ^AC^ (0.001)	0.133 ^BaC^ (0.000)
Rapeseed oil	0.049 ^ab^ (0.000)	0.051 ^cA^ (0.000)	0.052 ^acB^ (0.002)	0.052 ^bAB^ (0.001)
Sunflower oil	0.074 ^aAB^ (0.000)	0.077 ^ab^ (0.002)	0.078 ^A^ (0.001)	0.079 ^Bb^ (0.000)
EV Olive oil	0.178 ^a^ (0.001)	0.179 ^b^ (0.001)	0.180 ^c^ (0.001)	0.184 ^abc^ (0.002)
Index of thrombogenicity [IT]				
Palm oil	1.830 ^aA^ (0.007)	1.832 ^bB^ (0.029)	1.872 ^abC^ (0.014)	1.917 ^ABC^ (0.018)
Soybean oil	0.247 ^AB^ (0.002)	0.249 ^ab^ (0.003)	0.255 ^AaC^ (0.002)	0.261 ^BbC^ (0.001)
Rapeseed oil	0.088 ^aA^ (0.000)	0.091 ^bB^ (0.000)	0.094 ^abC^ (0.002)	0.095 ^ABC^ (0.001)
Sunflower oil	0.218 ^AB^ (0.000)	0.224 ^a^ (0.004)	0.227 ^AC^ (0.001)	0.231 ^BaC^ (0.001)
EV Olive oil	0.399 ^ab^ (0.003)	0.402 ^A^ (0.001)	0.405 ^ac^ (0.001)	0.414 ^bAc^ (0.003)
The hypocholesterolemic/hypercholesterolemic ratio [HH]				
Palm oil	1.056 ^aA^ (0.004)	1.054 ^bB^ (0.017)	1.033 ^abC^ (0.008)	1.009 ^ABC^ (0.010)
Soybean oil	5.629 ^AB^ (0.040)	5.583 ^a^ (0.075)	5.496 ^AC^ (0.036)	5.406 ^BaC^ (0.022)
Rapeseed oil	14.611 ^aA^ (0.034)	14.223 ^bB^ (0.035)	13.851 ^abC^ (0.353)	13.794 ^ABC^ (0.081)
Sunflower oil	9.048 ^AB^ (0.013)	8.777 ^a^ (0.161)	8.672 ^AC^ (0.046)	8.524 ^BaC^ (0.020)
EV Olive oil	4.531 ^ab^ (0.029)	4.504 ^ac^ (0.017)	4.477 ^d^ (0.012)	4.384 ^bcd^ (0.037)

Each data is calculated from six different chromatograms, data are mean (SD), common letters sharing a row denote significant differences between the different heating sequences of the same oil: ^abcd^: 0.05 < *p* ≤ 0.001; ^ABCDE^: *p* < 0.001 based on repeated measures with ANOVA and Bonferroni correction for multiple comparisons.

## Data Availability

The data presented in this study are available upon request to the corresponding author.

## Supplementary Materials

The following are available online at https://www.mdpi.com/article/10.3390/foods11020192/s1, Table S1: Changes in *trans* isomeric fatty acids in five different plant-based oils in fresh samples and after one (1H), five (5H) and ten heating sequences (10H).
