# Supplementary material for: Effects of Repeated Heating on Fatty Acid Composition of Plant-Based Cooking Oils
Source: Foods. 2022 Jan 12;11(2):192. doi: 10.3390/foods11020192 (PMC8774349; doi:10.3390/foods11020192)
Supplement: Supplementary file 1 [file foods-11-00192-s001.zip › foods-1531865-supplementary.pdf]

Table S1: Changes in *trans* isomeric fatty acids in five different plant-based oils in fresh samples and after one (1H), five (5H) and ten heating sequences (10H).

	**Palm oil**		**Soybean oil**		**Rapeseed oil**		**Sunflower oil**		**EV Olive oil**	
	**mean**	SD	**mean**	SD	**mean**	SD	**mean**	SD	**mean**	SD
**C18:1n-7/9*t***										
Fresh	**0.075**	0.003	**0.036**	0.002	**0.026**	0.009	**0.030**	0.003	**0.046**	0.022
1H	**0.099**	0.004	**0.084**	0.055	**0.066**	0.004	**0.038**	0.005	**0.069**	0.011
5H	**0.111**	0.003	**0.086**	0.025	**0.080**	0.010	**0.057**	0.003	**0.068**	0.002
10H	**0.136**	0.011	**0.091**	0.037	**0.102**	0.003	**0.070**	0.002	**0.103**	0.015
**C18:2n-6*tt***										
Fresh	**0.006**	0.000	**0.005**	0.001	**n.d.**	-	**0.004**	0.000	**n.d.**	-
1H	**0.005**	0.002	**0.004**	0.002	**n.d.**	-	**0.003**	0.001	**n.d.**	-
5H	**0.006**	0.001	**0.008**	0.001	**n.d.**	-	**0.007**	0.001	**n.d.**	-
10H	**0.007**	0.001	**0.011**	0.003	**0.002**	0.001	**0.011**	0.001	**0.002**	0.001
**C20:1n-9*t***										
Fresh	**0.024**	0.002	**0.050**	0.002	**0.023**	0.011	**0.018**	0.002	**0.010**	0.009
1H	**0.029**	0.004	**0.072**	0.029	**0.032**	0.006	**0.028**	0.003	**0.026**	0.011
5H	**0.043**	0.006	**0.073**	0.006	**0.053**	0.008	**0.041**	0.005	**0.065**	0.001
10H	**0.069**	0.008	**0.061**	0.005	**0.055**	0.027	**0.051**	0.012	**0.061**	0.006

Each data is calculated from six different chromatograms, data are **mean** (SD); n.d. denotes under detection limit
